# Histone methyltransferase SETDB1 promotes cells proliferation and migration by interacting withTiam1 in hepatocellular carcinoma

**DOI:** 10.1186/s12885-018-4464-9

**Published:** 2018-05-08

**Authors:** Yuqin Zhang, Jing Huang, Qisheng Li, Keli Chen, Yonghao Liang, Zetao Zhan, Feng Ye, Wen Ni, Longhua Chen, Yi Ding

**Affiliations:** Department of Radiation Oncology, Nanfang Hospital, Southern Medical University, Guangzhou, Guangdong Province China

**Keywords:** Tiam1, SETDB1, Hepatocellular carcinoma

## Abstract

**Background:**

SETDB1 is a histone H3K9 methyltransferase, which plays a significant role in the occurrence and progression of tumors. Previous studies have confirmed that T-lymphom invasion and metastasis gene (Tiam1) is a protein associated with the metastasis of hepatocellular carcinoma (HCC); however, we have not yet been successful in elucidating the specific mechanism of HCC.

**Methods:**

Yeast two-hybrid test was conducted to screen proteins that interacted with Tiam1 gene. Glutathione-S-transferase (GST) pull-down and crosslinking-immunoprecipitation (CLIP) assays were performed to determine whether SETDB1 can interact with Tiam1 gene. A series of related experiments were performed to explore role of SETDB1 on cell proliferation, migration, and invasion in HCC. Recovery experiment was performed to investigate the effect of Tiam1 knockdown on cell proliferation and migration, which was caused by SETDB1 overexpression in HCC cells. The expression of SETDB1 was frequently upregulated in HCC tissues and positively correlated with Tiam1.

**Results:**

GST pull-down and CLIP assays were performed to elucidate the interaction between SETDB1 and Tiam1. Cell proliferation, migration, and epithelial mesenchymal transformation (EMT) in HCC cells was promoted with the overexpression of SETDB1. Following the knockdown of Tiam1 gene, the effect of SETDB1 on cell proliferation and migration was reversed in HCC cells. The expression of SETDB1 was frequently up-regulated in HCC tissues, and it was positively correlated with Tiam1 gene.

**Conclusions:**

Ours is the first study to prove that SETDB1 promotes the proliferation and migration of cells by forming SETDB1-Tiam1 compounds. We found that SETDB1-Tiam1 compounds were involved in a novel pathway, which regulated epigenetic modification of gene expression in HCC samples.

**Electronic supplementary material:**

The online version of this article (10.1186/s12885-018-4464-9) contains supplementary material, which is available to authorized users.

## Background

Hepatocellular carcinoma (HCC) is one of the most common malignancies in humans [1]. The rate of intrahepatic and extrahepatic metastases is high because of poor prognosis of HCC; moreover, the recurrence rate of HCC is high [2]. Metastasis usually occurs in patients with advanced HCC, so it is important to develop new therapeutic targets for successful intervention. Metastasis is a complex process; metastatic potential of HCC cells is governed by cell intrinsic identities and external micro-environmental factors [3]. However, we still need to elucidate the underlying molecular mechanisms that mediate metastatic cascade. By further elucidating the molecular mechanism, we can promote the development of effective metastasis-targeted therapy. This would improve the quality of life and survival of patients with HCC.

Previous studies have shown that metastasis of HCC occurs due to Tiam1 gene, which is a member of Dbl gene family that governs guanine nucleotide-exchange factors (GEFs) [4]; however, the underlying molecular mechanism is hardly known. We conducted a more in-depth study to further investigate the underlying mechanism of HCC.

In this study, yeast two-hybrid assay was performed to screen the interaction of proteins with Tiam1 gene. We acquired, sequenced, and analyzed 24 positive clones from NCBI database. Finally, we identified six proteins, namely, OSBPLlA, ZNF307, FNDC3B, SRSF5, SYCPl, and SETDB1. After literature mining and bioinformatics analysis, we selected SETDB1 for further verification study.

The protein SETDB1, which is also known as KMT1E, is an H3K9 methyltransferase (HMT). Its multiple functional domains are located on chromosome 1q21. One of the major functions of HMT is covalent histone modification, which involves following processes: acetylation (Ac), methylation (Me), phosphorylation, ubiquitination, and sumoylation. These processes were associated with the development and progression of various tumors [5]. According to the report that loss of H3K9Me2 is related with poor prognosis of both prostate and kidney cancer [6, 7]. In addition, H3K9Me3 also serves as a diagnostic marker for the recurrence and distant metastasis of various cancers [8, 9]. Furthermore, SETDB1 is associated with transcriptional inhibition of euchromatin, while another H3K9 methyltransferase SUV39H1 is mainly responsible for the high expression of structural pericentromeric heterochromatin, which functions as an oncogene in the metastasis of HCC cancer [10, 11]. A lot of studies have clearly stated that tumorigenesis is promoted by HMTs, including Suv39h1 and G9a (EHMT2) in the past few decades, [11]. By suppressing the expression of G9a and Suv39h1, cell growth is inhibited and lung epithelial cells are transformed in prostate cancer patients [12, 13]. These researches support the hypothesis that aberrant histone methylation leads to the activation or repression of certain important genes during tumorigenesis. However, SETDB1, which serves as H3K9 methyltransferase, has been rarely associated with carcinogenesis and migration of liver. Therefore, we need to comprehensively investigate functional and pathological roles of SETDB1 in HCC patients.

Preliminary, Tiam1 was associated with the metastasis of liver cancer. Herein, it was found that SETDB1 is a crucial oncogene in the metastasis of HCC. In addition, we proved that cell invasion and metastasis was enhanced when SETDB1 cooperated with Tiam1. These observations indicate there was a novel pathway to regulate epigenetic modification of genes during HCC metastasis.

## Methods

### Cell lines and culture conditions

HCC cell lines QGY-7701(Catalogue number:TCHu42), Bel-7402(Catalogue number:TCHu10) and HCCLM3(Catalogue number:TCHu94)were purchased from Shanghai Cell Bank, Chinese Academy of Sciences, MHCC97L cell line(Product number: BNCC337741)was purchased from Bei Na Chuanglian Institute of Biotechnology of Beijing, china. All cell lines were cultured in DMEM or RPMI-1640 medium, which contained 10% FBS (Gibco, USA). As described previously, cells were cultured at 37 °C under 5% CO_2_ [4].

### Collection of specimens

Primary HCC specimens and corresponding adjacent non-tumorous (NT) liver specimens were collected from Nanfang Hospital, Southern Medical University in China. A written informed consent letter was obtained from every study participant. The collection of specimens was approved by the Ethics Committee of Nanfang Hospital, Southern Medical University, Guangdong Province, China.

### RNA isolation, reverse transcription and qRT-PCR

Total RNA was extracted with Trizol Reagent (Invitrogen,USA) according to manufacturer’s protocol. In the PrimeScript RT reagent kit (TaKaRa,Japan), total RNA was used as a template for the production of cDNA. To analyze the expression of mRNA, qRT-PCR was performed with SYBR Green qRT-PCR master mix (TaKaRa,Japan)on a Stratagene Mx3005P qRT-PCR system. The target genes of RT-qPCR were calculated with relative quantification (2^−ΔΔCt^) method, which was normalized to GAPDH.

### CCK8 assays

Cells were plated in 96-well plates; a final density of 1 × 10^3^ cells was maintained per well. After incubating cells for 24 h, 2-(2-methoxy-4-nitrophenyl)-3- (4-nitrophenyl)-5-(2,4-disulfonic acid benzene)-2H-tetrazole monosodium salt (CCK8) assay was performed by adding 10 μl of reagents (Beyotime, China) to plates. After incubating cells for 1 h, the absorbance of each well was measured with a microplate reader at 570 nm. The absorbance of cell culture was determined continuously for the next six days. All experiments were performed in triplicate.

### Clonogenic survival assays

Clonogenic survival assays were carried out as described previously [4].

### Cell migration and invasion assays

Cell migration and invasion assays were performed in Transwell chambers (Corning Costar, USA). Matrigel (BD Bioscience, USA) was added to the chambers in which invasion assay was carried out. Before performing the assay, cells were serum-starved for 24 h. Migration assay was performed as follows: 1× 10^5^ tumor cells were plated into the upper chamber with 0.1% fetal bovine serum (FBS). Then, 10% FBS was used as a chemo-attractant, which was added to the lower chamber. Invasion assay was performed as follows: cells were seeded on filters, which were coated with 20–50 μg/cm^2^ of reconstituted Matrigel (BD Bioscience, USA) on basement membranes. After incubating cells for 24 h at 37 °C, we used cotton swabs to remove cells that had not migrated or invaded from the top surface of filters. After the cells migrated or invaded into the bottom surface, they were fixed with 100% methanol and stained with 0.5% crystal violet. Permeating cells were observed under a microscope at × 200 magnification; these cells were graphed in six randomly selected fields. The experiment was repeated thrice independently.

### Antibodies and western blotting

Western blot analysis was performed according to a procedure described previously [14].

### Yeast two-hybrid

To determine the expression of Tiam1 in yeast cells, a recombinant plasmid pGBKT7-Tiam1/C1199 was fused by recombinant gene technique. The code domain sequence of Tiam1/C1199 was amplified from a commercial Tiam1 cDNA clone, which was then cloned into pGBKT7 vector and confirmed by sequencing. The yeast strain AH109 cells were transiently transformed with pGBKT7-Tiam1/C1199 plasmid. Western blot was performed to confirm whether Tiam1 protein can be expressed normally in *Saccharomyces cerevisiae* without toxicity or autoactivation.

### GST pull-down assay

Inoculate several colonies containing pGEX-4 T-1-Tiam1-PCER, C685, C751, C1199, and control. The recommended proteins were expressed in transformed cells of E.coli. These proteins were then purified. We successfully detected fusion proteins of Tiam1, which were labeled with GST. The purified protein SETDB1 was acquired with TNT® Quick Coupled Transcription/Translation Systems (Promega,USA). The interaction between Tiam1 and SETDB1 was detected and validated in vitro with GST pull-down assay(The detailed procedures could be seen in Additional file 1).

### Cross-linking immunoprecipitation

Some different epitope-labeled candidate proteins (Flag and HA) and the recombinant expression vector Tiam1 were constructed by recombinant DNA technology. The recombinant plasmid had different epitope labeling. The recombinant plasmid Tiam1-C1199 was co-transfected into human embryonic kidney cells HEK293T. Cells were fixed at room temperature for 10 min with 10 ml of 1% formaldehyde in phosphate buffered saline (PBS). Then, these cells were sonicated to isolate total cellular protein. Protein, antibody, and protein G were incubated together. Protein samples were analyzed by western blot technique: the presence of proteins was detected with FLAG and HA antibodies in order to determine whether Tiam1 truly interacted with SETDB1 protein.

### Establishment of stable cell lines

For the knockdown or overexpression of SETDB1, we purchased lentivirus vector and control vector from Genechem (Shanghai, China). Stably knockdown or overexpression of SETDB1 cells lines were constructed with lentiviruses transfection. Finally, the expression of SETDB1 was quantified by performing western blot analysis.

### Xenograft studies

The stably knockdown or overexpression of SETDB1cells were suspended in 25 μL PBS. These cells were injected into the left liver lobe of male BALB/C nude mice aged 6-8 weeks. These cells were purchased from the Medical Experimental Animal Center of Guangdong Province in China. After six weeks, phenobarbital sodium was used to euthanize mice. Then, liver and lungs were removed from the euthanized mice. Xenograft tumor assays were performed as follows: 1 × 10^6^ cells were injected subcutaneously into the flanks of nude mice, which were 4-6 weeks old. When the tumors could be detected, we measured the size of tumors every three days with a slide caliper for 21 days. Animal experiments were performed by strictly adhering to the Regulations for the Administration of Affairs Concerning Experimental Animals. The Institutional Animal Care and Use Committee approved all the procedures performed on animals in this study.

### Statistical analysis

Data were expressed as mean ± standard deviation (SD), and a *P value of* < 0.05 was considered to be statistically significant in all the experiments. Results were analyzed by performing ANOVA or a two-tailed Student’s t-test. Statistical analysis was performed with SPSS 13.0 software (SPSS; North Chicago, IL, USA). Western blot results were quantified with Bio-Rad lab image software. In this study, all the experiments were performed in triplicates.

## Results

### Screening proteins interacting with Tiam1 by yeast two-hybrid

pGBKT7-Tiam1/C1199 was used as a bait to screen the “Universal Human Mate & Plate Library”. A total of 24 positive clones were obtained, sequenced, analyzed in NCBI database. Then, they were matched exactly with six known proteins: OSBPL1A, ZKSCAN4 (also known as ZNF307), FNDC3B, SRSF5, SYCP1, and SETDB1 (Additional file 2: Figure S1, Additional file 3: Figure S2, Additional file 4: Figure S3 and Additional file 5: Figure S4).

### SETDB1 interacts with Tiam1 both in vivo and vitro

**Fig. 1:**
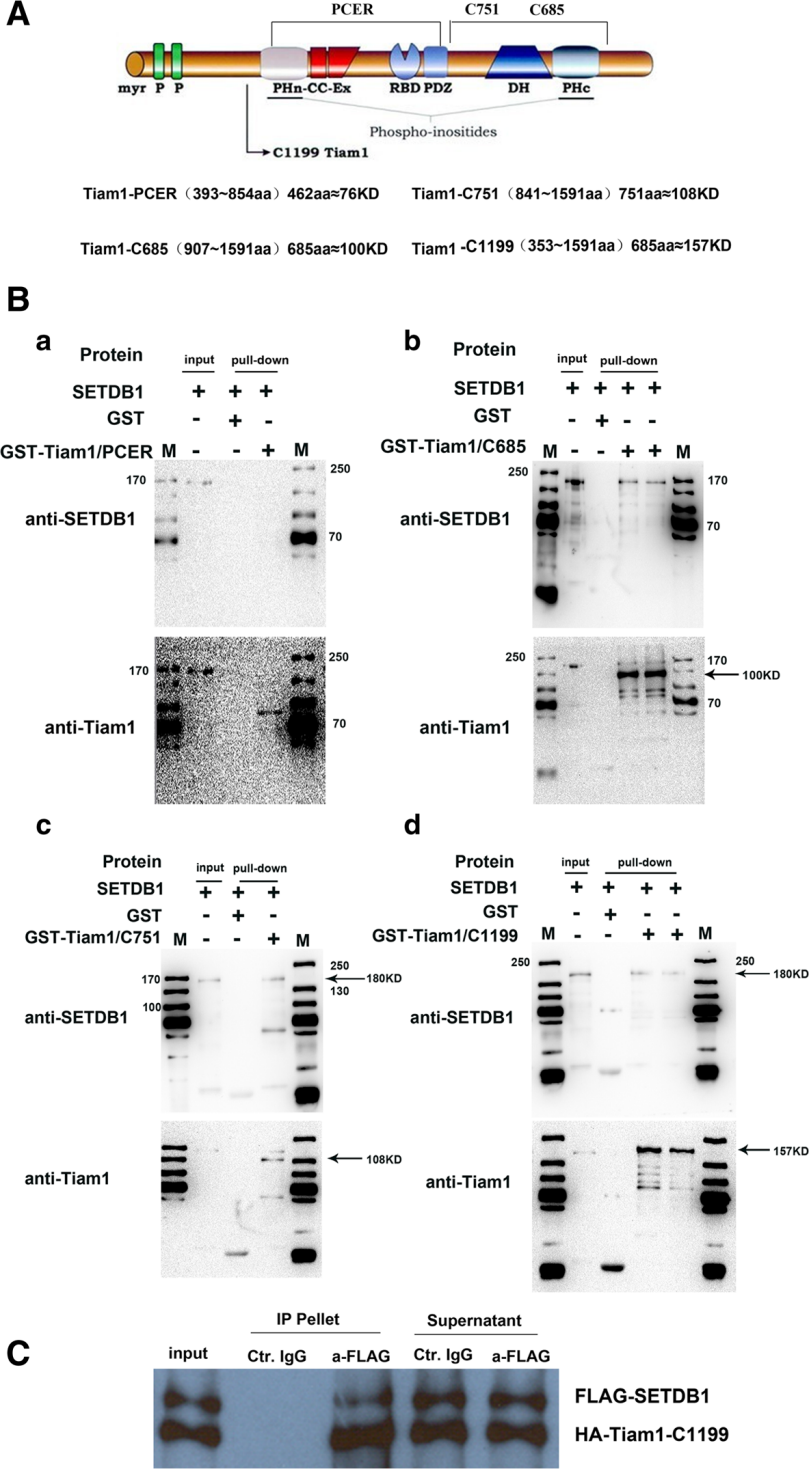
Tiam1 could interacted with SETDB1directly by GST- pull down and Cross-linking assays. **A** Schematic representation of the domain structure of Tiam1. PCER; C685;C751 and C1199 were four truncations constructed containing different domains. **B** Interaction sites was verified by GST-pull down and westernblot assays. Proteins pulled down by agarose beads with GST tag further were detected by westernblot, (a) purified Tiam1-PCER protein labelled with GST was co-incubated with purified SETDB1 and detected by westernblot after elution, as shown in figure.a, Tiam1-PCER could be detected but not STEDB1, indicating the Tiam1-PCER fragment has no binding sties with SETDB1 (b,c,d) purified Tiam1-C685 protein was obtained as indicated, as shown in above figures, Tiam1-C685, C751 and C1199 could be detected as well as STEDB1, indicating the these fragments have binding sties with SETDB1. **C** Cross-linking assay confirmed Tiam1could interacted with SETDB1

Mass spectrometry results were identified in vivo and in vitro by GST pull-down and co-immunoprecipitation. The possible structural domains of SETDB1 and Tiam1 were detected with bioinformatics and literature analysis. Four Tiam1 truncation mutants were constructed: GST-Tiam1-PCER, GST-Tiam1-C685, GST-Tiam1-C751, and GST-Tiam1-C1199. After purifying different Tiam1 and SETDB1 proteins, we screened and incubated them with agarose beads; these beads were marked with GST (Additional file 6: Figure S5). Results indicate that SETDB1 could be detected when incubated with following proteins: GST-Tiam1-C685, GST-Tiam1-C751, and GST-Tiam1-C1199; however, the protein GST-Tiam1-PCER was not useful for detection of SETDB1. This indicates that the interaction domain was located at PDZ/DH/PHc region (Fig. 1a and b). Thus, the interaction between SETDB1 and Tiam1 was confirmed in vitro.

The interaction between Tiam1 and SETDB1, which were obtained from exogenous transfectants, was verified by co-immunoprecipitation. The proteins Tiam1 and Flag-SETDB1 could be detected by corresponding antibody when we performed immunoprecipitation (IP) with anti-flag antibody. This proves that a strong interaction existed between Tiam1 and SETDB1 (Fig. 1c).

### SETDB1 promotes the proliferation of cells in HCC both in vitro and in vivo

**Fig. 2:**
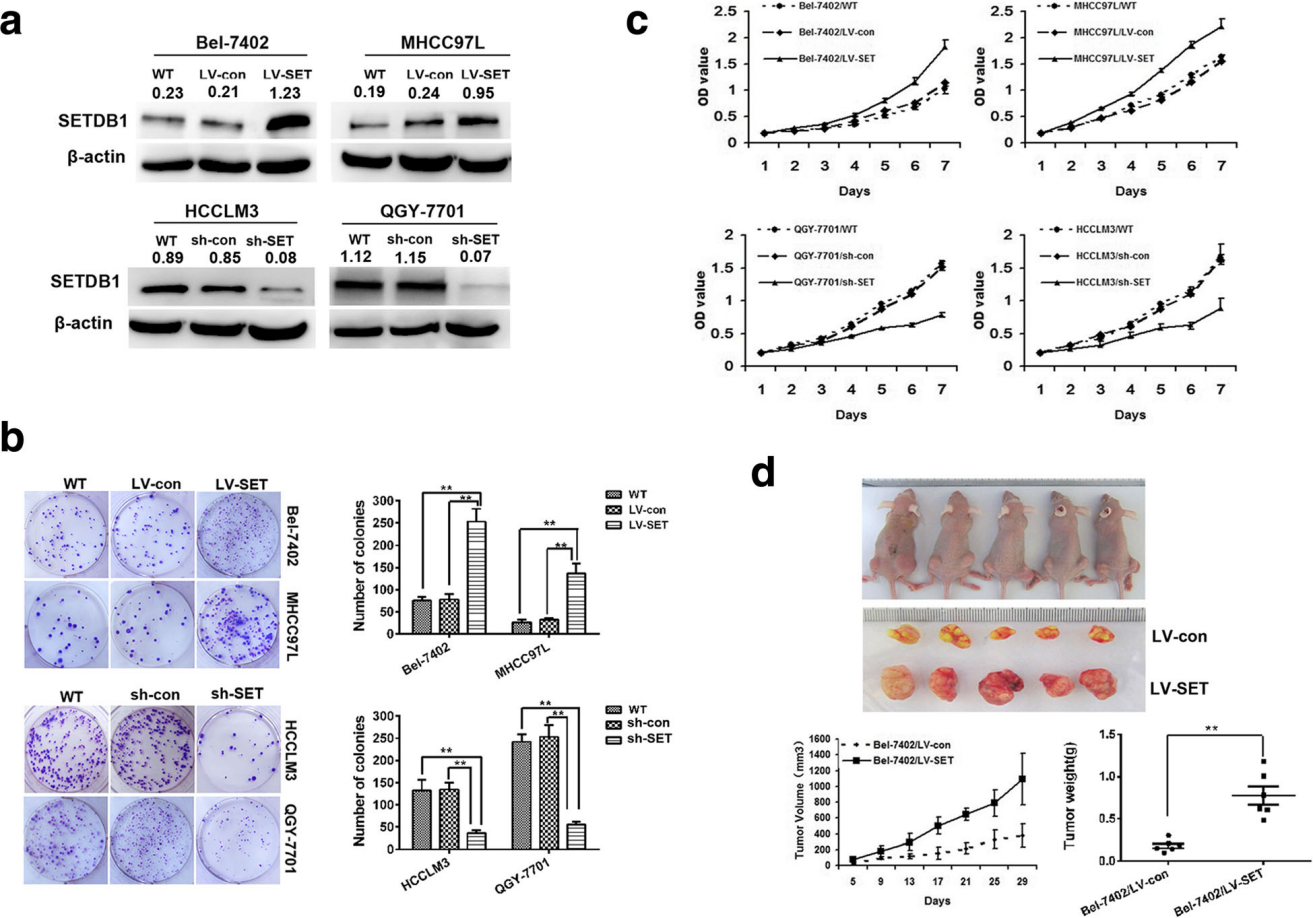
SETDB1 promoted cell proliferation both in vivo and vitro in HCC. **a** The expression of SETDB1 was detected by westernblot after transfecting with overexpression and knockdown virus.** b** Overexpression of SETDB1 could promote cellsproliferation ability was detected by CCK8 and Plate clony formation assays. ** *P*<0.01. **c** knockdown of SETDB1 could inhibit cellsproliferation ability was detected by CCK8 and Plate clony formation assays. ** *P*<0.01. **d** SETDB1 could promote HCC cell proliferation in vitro by xenografts experiments. ** *P*<0.01

Tiam1 always serves as an oncogene in tumorigenesis. A series of research studies have established the interaction between Tiam1 and SETDB1. To determine the effect of SETDB1 on cell growth, Bel-7402 and MHCC97L cells were transfected with the stable lentivirus SETDB1. The knockdown and overexpression of SETDB1 was verified by performing western blot analysis (Fig. 2a). By performing CCK8 proliferation assay and plate colony formation assay, we confirmed that proliferation and colony formation ability of HCC cells could be promoted with an overexpression of SETDB1; this effect was not observed in control cells. However, an opposite effect was observed in HCC cell lines following the knockdown of SETDB1 (Fig. 2b and c).

An orthotopic subcutaneous tumor model was used to further verify the effect of SETDB1 on HCC tumorgenesis. Compared to the control, the size of tumor increased significantly when HCC cells were subjected to an overexpression of SETDB1 in vivo. In contrast, an opposite effect was observed following the knockdown of SETDB1 (Fig. 2d). This observation confirmed the results in vitro. The results indicate that SETDB1 functioned as an oncogene, which was vital for the proliferation of HCC.

### SETDB1 promotes the metastasis of HCC cells in vitro and in vivo

**Fig. 3:**
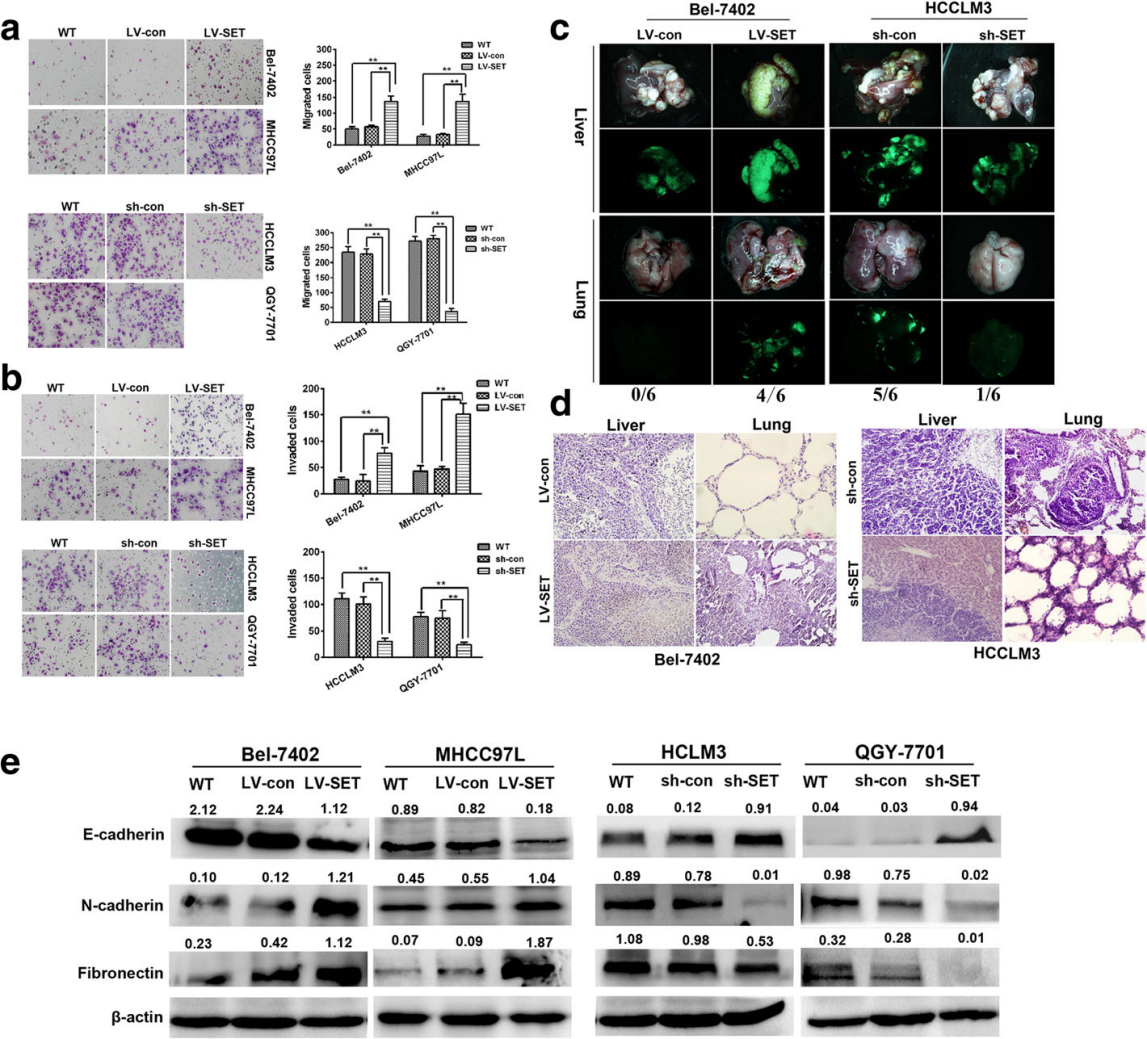
SETDB1 promoted cell metastasis both in vivo and vitro in HCC. **a** and **b** Transwell and Boyden assays indicate SETDB1 could enhance cell migration and invasion. The invasive cells were stained and counted under microscope at 24–30 h after reseeding. Original magnification, × 400. ** *P*<0.01, as compared LV-SET with WT and LV-con groups, or LV-SET with WT and LV-shcon groups. **c** SETDB1 significantly promoted HCC tumorigenicity in vivo as demonstrated by an orthotopic tumor implantation experiment in nude mice. **d** SETDB1 increased lung metastasis in an orthotopic tumor implantation model in nude mice. Hematoxylin and eosin staining confirming the formation of HCC tumor foci in the lungs. **e** SETDB1 modulates EMT-related genes expression

Previous studies have confirmed that Tiam1 is essential to promote the invasion of HCC cells. Transwell and Boyden Chambers’ assays prove that the penetration of cells increased in membranes following the overexpression of SETDB1 in Bel-7402 and MHCC97L cells. Meanwhile, an opposite effect was observed following the knockdown of SETDB1 in HCC cell lines. Migration and invasion assays proved that compared to control cells, the motility and invasiveness of Bel-7402 and MHCC97L cells was remarkably enhanced with the overexpression of SETDB1 (Fig. 3a and b). Moreover, our study indicates that the expression of E-cadherin decreases while the expression of N-cadherin increases due to the overexpression of SETDB1. Moreover, cell epithelial-mesenchymal transition is induced with an overexpression of SETDB1 (Fig. 3e). This indicates that SETDB1 can promote the migration and invasion of HCC cells in vitro.

To further verify the effect of SETDB1on HCC invasion,we orthotopicly transplanted cells to the left hepatic lobe. Compared to non-target shRNA control, lung metastasis was significantly increased with an overexpression of SETDB1 in Bel-7402 cells (Fig. 3c and d). This indicates that SETDB1 acted as an oncogene, which was essential for the metastasis of HCC.

### Tiam1 knockdown reverses the effect of SETDB1 on cell proliferation and migration in HCC

**Fig. 4:**
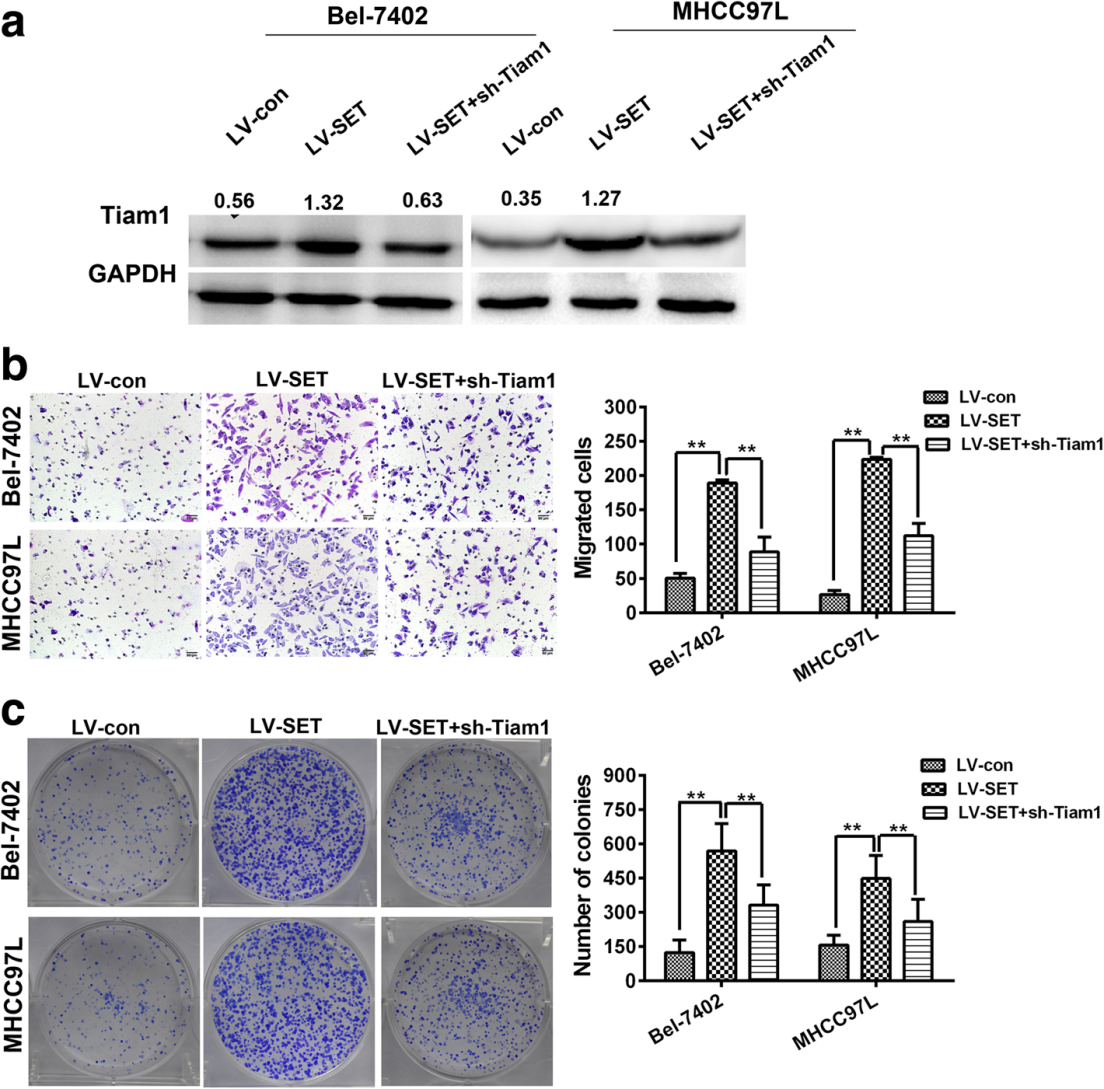
Tiam1 is a functional target of SETDB1. **a** The expression of Tiam1 was identified by westernblot analysis. **b** Knockdown of Tiam1 could offset the effect of SETDB1 on cell migration ability by transwell assay. **c** Knockdown of Tiam1 could decrease the number of colonies caused by SETDB1

**Fig. 5:**
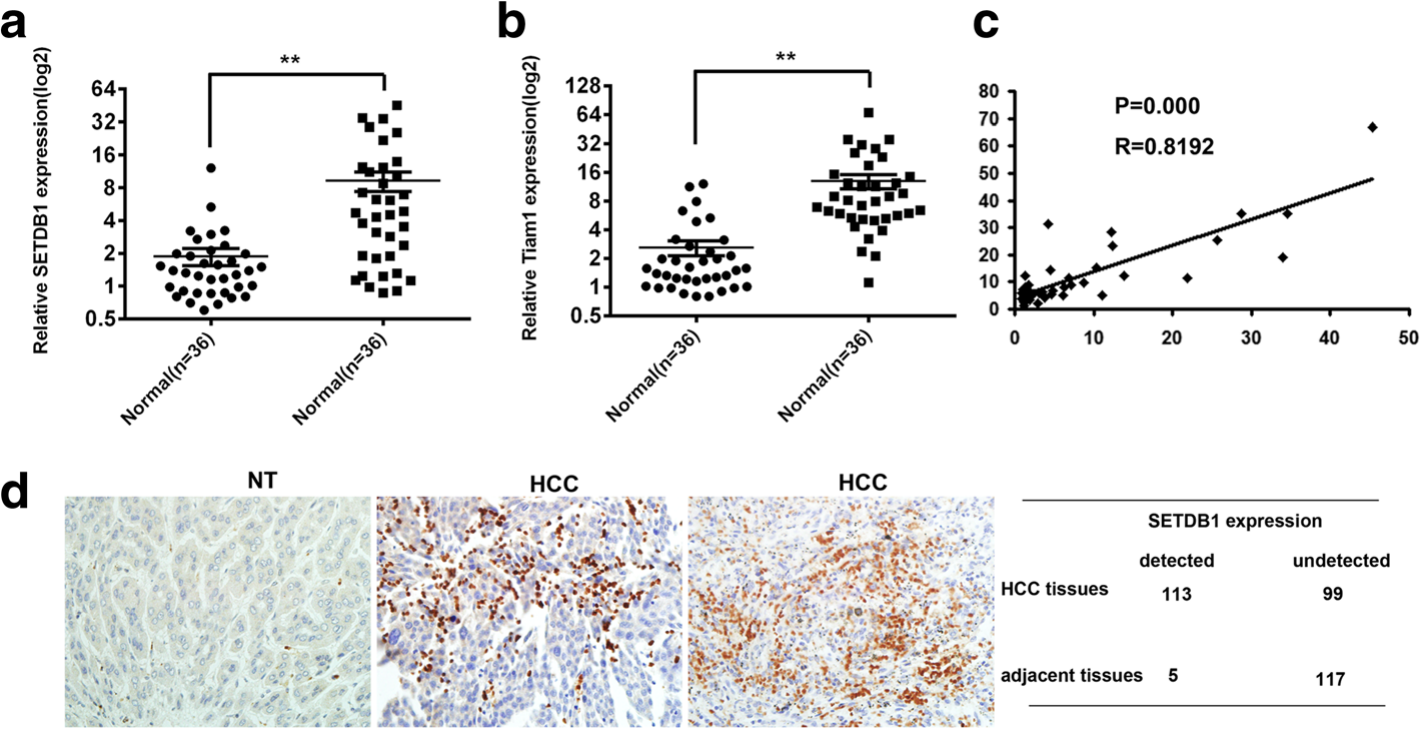
SETDB1 expression is associated with Tiam1expression in HCC cancer. **a** The expression of SETDB1 and Tiam1 in HCC biopsy samples and control normal samples detected by qRT-PCR. **b** SETDB1correlates positively with Tiam1 in HCC tissue samples. **c** The expression of SETDB1 in HCC tissues and adjacent normal tissue were detected by IHC

The knockdown virus Tiam1was transfected into overexpressed cells of SETDB1; these cells were identified by western blot (Fig. 5a). Colony formation and transwell assays were conducted. The results indicate that Tiam1 knockdown can offset the effect of SETDB1, which is a functional target of SETDB1 on HCC (Fig. 4b and c).

### SETDB1 is frequently upregulated in HCC tissues and positively correlated with Tiam1

The expression of SETDB1 and Tiam1 were examined in 36 HCC specimens and matched normal tissues. Compared to normal tissues, the average expression of SETDB1 and Tiam1 was significantly higher in HCC specimens (Fig. 5a and b). Moreover, the expression of SETDB1 was correlated with Tiam1 positively (Fig. 5c).

In tissue sections of HCC and non-tumor (NT) liver, the expression of SETDB1 was further validated by IHC. Furthermore, SETDB1 can hardly be detected in normal liver tissues: SETDB1 was detected in 53.3% (113/212) of primary HCC cases by IHC. The results indicate that compared to adjacent hepatocytes, the expression of SETDB1 was frequently up-regulated in the nucleus of HCC cells (Fig. 5d).

## Discussion

The gene Tiam1 is is a member of the guanine nucleotide exchange factor (GNEF) family which is located on the human chromosome 21. Previous studies showed that the expression of Tiam1 gene is up-regulated in following types of carcinomas: T lymphoma, B lymphoma, pancreatic carcinoma, gastric cancer, breast cancer, and lung cancer [15–18]. The expression of Tiam1 was regulated by epigenetic mechanisms [19–21]. Previous studies have reported that Tiam1 gene interacts with many proteins, such as IB2/JIP2, nm23H1, Arp2/3, CD44, PAR, c-myc, EphB2, and Par-3. Therefore, Tiam1 gene is involved in many biological processes [22–25]. However, researchers have not been successful in elucidating the inherent molecular mechanisms associated with HCC cells; these mechanisms are apparently mediated by Tiam1.

In this study, yeast two-hybrid method was used to discover and validate the interaction between candidate proteins and Tiam1. The results indicate that OSBPLlA, ZNF307, FNDC3B, SRSF5, SYCPl, and SETDB1 were candidate proteins. The interaction between Tiam1 and candidate proteins was confirmed by cross-linking IP.

The candidate protein SETDB1 is a major histone methyltransferase (HMT), which is associated with transcription repression through H3K9me3 [10].

The candidate protein SETDB1 plays an significant role on transcriptional repression of euchromatin, the activity of which is important for the maintenance of embryonic stem cell state by repressing lineage-specific gene expression [10, 26, 27]. Previous studies have reported the SETDB1 overexpression in patients was detected in certain tumors such as melanoma and lung cancer [28, 29]. Previous research studies have reported SETDB1 accelerates tumorigenesis by regulating WNT signaling pathway [29]. Recently, SETDB1 was reported as a novel oncogene in zebrafish melanoma model [30]. However, we have not completely explored tumorigenesis mechanism of SETDB1, which is associated with the growth and metastasis of HCC tumor.

The interaction between Tiam1 and SETDB1 was determined by performing crosslinking IP and GST-pull down assays. It is important to note that SETDB1 is a major histone methyltransferase (HMT). During tumorgenesis, critical genes are activated or repressed by aberrant histone methylation. Moreover, H3K9Me3 serves as a significant diagnostic marker of recurrence and distant metastasis in cancer patients [8, 9].

We investigated whether SETDB1 acts as an oncogene in patients with HCC. The proliferation, migration, and invasion of HCC cells were promoted with the overexpression of SETDB1. Compared to adjacent liver tissues, the expression of both SETDB1 and Tiam1 was unregulated in HCC samples; moreover, the expression of SETDB1 and Tiam1 was positively correlated with HCC samples. The effect of SETDB1 was restored on cells following the knockdown of Tiam1. In this study, a complex was formed following the interaction of SETDB1 with Tiam1. This indicates that SETDB1 functions as an oncogene in HCC cells.

## Conclusions

In our work we confirm that proliferation, migration, and invasion of HCC cells could be promoted with the overexpression of SETDB1. Compared to adjacent liver tissues, the expression of both SETDB1 and Tiam1 was up-regulated in HCC samples; moreover, there was positive correlation between the expression of SETDB1 and Tiam1. In our study, SETDB1 interacted with Tiam1 to form a complex. This establishes that SETDB1 functions as an oncogene in HCC samples. These results support a novel link between SETDB1 and Tiam1; moreover, the complex SETDB1-Tiam1 is associated with the growth and metastasis of HCC tumor. They also suggest that SETDB1-Tiam1 complex is involved in a novel pathway, which regulates epigenetic modification of gene expression in HCC.

## Additional files

Additional file 1: The detailed procedures of GST-pull down assay (DOC 43 kb)

Additional file 2: **Figure S1.** A. Confirmation of the BD-hTiam1-C1199 clones by PCR M:5000bpDNA; Ladder one to three: three BD-hTiam1-C1199 clones amplified by hTiam1-C1199 forward and reversed primers; **+ve:** commercial human Tiam1 clone amplified by hTiam1-C1199 forward and reversed primers. B Confirmation of the BD-hTiam1-C1199 clones by enzyme digestion. M:1 kb DNA; Ladder one to three: three BD-hTiam1-C1199 clones digested with NdeI and XmaI.). C Partial sequencing map of Tiam1-C1199 clone. (TIF 13173 kb)

Additional file 3: **Figure S2.** (A) Expression of the BD-hTiam1-C1199 clones detected by anti-c-Myc antibody. (ladder1:BD alone, ladder1 2: BD-p53 fusion protein, ladder3-5: BD-hTiam1-C1199 clone ^#^1-3). (B)Testing for autoactivation by plating AH109 cells co-transformed with the bait plasmid and the AD vector on SCM-2 and -3 plates. SCM-2, SCM plate lacking leucine and tryptophan; cell growth shows the successful co-transformation; SCM-3, SCM plate lacking leucine, tryptophan and histidine; no cell growth shows no autoactivation of transcription by the BD-hTiam1-C1199 fusion protein. (a. SCM/−Trp-Leu (− 2) Plate;b. SCM/−Leu-Trp-His (− 3) Plate). (TIF 9682 kb)

Additional file 4: **Figure S3.** (A) Positive colonies were verified by re-hybrid assay. (B) Summary of Tiam 1 yeast two hybrid results. Rating ≥ 2: Positive candidates; =1: possible candidates (some confirmed); =0:negative candidates (interacting with BD). ‘0’: no colony on SCM-Trp-Leu-His (− 3) plate; ‘+’: small sized and/or red colonies on − 3 only; ‘++’: normal sized white colonies on − 3; ‘+++’: normal sized white colonies on − 3 and small sized and/or red colonies on SCM-Trp-Leu-His-Ade (− 4) plate;++++’ normal sized white colonies on − 4. (TIF 10966 kb)

Additional file 5: **Figure S4.** Yeast two-hybrid Screening for proteins interactions with Tiam1 and confirmed by Sequencing and blast NCBI database. A Screening positive clones obtained by using a different degree defective media. B SETDB1was one of the possible protein interaction with Tiam1 by blast NCBI database. C The sequencing of one positive clone screened out. (TIF 10416 kb)

Additional file 6: **Figure S5.** (A) IPTG successfully induction of four different domains as named of Tiam1 and confirmed by Coomassie brilliant blue staining. (B) Four different domains of Tiam1 were purified by agarose beads with GST tag. (C) Verify the expression of SETDB1by TNT transcription and translation kit in vitro. (TIF 12668 kb)

## Abbreviations

CCK8: 2-(2-methoxy-4-nitrophenyl)-3- (4-nitrophenyl)-5-(2,4-disulfonic acid benzene)-2H-tetrazole monosodium salt
CLIP: Crosslinking immunprecipitation
EMT: Epithelial mesenchymal transformation
GST: Glutathione-S-transferase
HCC: Hepatocellular carcinoma
HMT: Histone methyltransferase
Tiam1: T-lymphom invasion and metastasis gene

## References

[1] Jemal A (2011). Global cancer statistics. CA Cancer J Clin.

[2] Budhu A (2006). Prediction of venous metastases, recurrence, and prognosis in hepatocellular carcinoma based on a unique immune response signature of the liver microenvironment. Cancer Cell.

[3] Fidler IJ (2003). The pathogenesis of cancer metastasis: the 'seed and soil' hypothesis revisited. Nat Rev Cancer.

[4] Huang J (2013). Tiam1 is associated with hepatocellular carcinoma metastasis. Int J Cancer.

[5] Shinjo K, Kondo Y (2012). Clinical implications of epigenetic alterations in human thoracic malignancies: epigenetic alterations in lung cancer. Methods Mol Biol.

[6] Ellinger J (2010). Global levels of histone modifications predict prostate cancer recurrence. Prostate.

[7] Seligson DB (2009). Global levels of histone modifications predict prognosis in different cancers. Am J Pathol.

[8] Park YS (2008). The global histone modification pattern correlates with cancer recurrence and overall survival in gastric adenocarcinoma. Ann Surg Oncol.

[9] Song JS (2012). Global histone modification pattern associated with recurrence and disease-free survival in non-small cell lung cancer patients. Pathol Int.

[10] Schultz DC (2002). SETDB1: a novel KAP-1-associated histone H3, lysine 9-specific methyltransferase that contributes to HP1-mediated silencing of euchromatic genes by KRAB zinc-finger proteins. Genes Dev.

[11] Chen MW (2010). H3K9 histone methyltransferase G9a promotes lung cancer invasion and metastasis by silencing the cell adhesion molecule ep-CAM. Cancer Res.

[12] Kondo Y (2008). Downregulation of histone H3 lysine 9 methyltransferase G9a induces centrosome disruption and chromosome instability in cancer cells. PLoS One.

[13] Watanabe H (2008). Deregulation of histone lysine methyltransferases contributes to oncogenic transformation of human bronchoepithelial cells. Cancer Cell Int.

[14] Zhang Y (2015). MiR-20a induces cell Radioresistance by activating the PTEN/PI3K/Akt signaling pathway in hepatocellular carcinoma. Int J Radiat Oncol Biol Phys.

[15] Chen JS (2012). Expression of T-cell lymphoma invasion and metastasis 2 (TIAM2) promotes proliferation and invasion of liver cancer. Int J Cancer.

[16] Zhu JM, Yu PW (2013). Downregulation of Tcell lymphoma invasion and metastasisinducing factor 1 induces cytoskeletal rearrangement and inhibits the invasive capacity of gastric cancer cells. Mol Med Rep.

[17] Liu S (2014). Expression of Tiam1 predicts lymph node metastasis and poor survival of lung adenocarcinoma patients. Diagn Pathol.

[18] Zhao L (2011). Overexpression of T lymphoma invasion and metastasis 1 predict renal cell carcinoma metastasis and overall patient survival. J Cancer Res Clin Oncol.

[19] Hu J (2015). The downregulation of MiR-182 is associated with the growth and invasion of osteosarcoma cells through the regulation of TIAM1 expression. PLoS One.

[20] Li Z (2015). By downregulating TIAM1 expression, microRNA-329 suppresses gastric cancer invasion and growth. Oncotarget.

[21] Whalley HJ (2015). Cdk1 phosphorylates the Rac activator Tiam1 to activate centrosomal Pak and promote mitotic spindle formation. Nat Commun.

[22] Wang S (2012). Tiam1 interaction with the PAR complex promotes Talin-mediated Rac1 activation during polarized cell migration. J Cell Biol.

[23] Rajagopal S (2010). Scaffold proteins IRSp53 and spinophilin regulate localized Rac activation by T-lymphocyte invasion and metastasis protein 1 (TIAM1). J Biol Chem.

[24] Tanaka M (2004). Tiam1 mediates neurite outgrowth induced by ephrin-B1 and EphA2. EMBO J.

[25] Otsuki Y (2001). Tumor metastasis suppressor nm23H1 regulates Rac1 GTPase by interaction with Tiam1. Proc Natl Acad Sci U S A.

[26] Bilodeau S (2009). SetDB1 contributes to repression of genes encoding developmental regulators and maintenance of ES cell state. Genes Dev.

[27] Yuan P (2009). Eset partners with Oct4 to restrict extraembryonic trophoblast lineage potential in embryonic stem cells. Genes Dev.

[28] Rodriguez-Paredes M (2014). Gene amplification of the histone methyltransferase SETDB1 contributes to human lung tumorigenesis. Oncogene.

[29] Sun QY (2015). SETDB1 accelerates tumourigenesis by regulating the WNT signalling pathway. J Pathol.

[30] Ceol CJ (2011). The histone methyltransferase SETDB1 is recurrently amplified in melanoma and accelerates its onset. Nature.

