# Supplementary material for: Histone methyltransferase SETDB1 promotes cells proliferation and migration by interacting withTiam1 in hepatocellular carcinoma
Source: BMC Cancer. 2018 May 8;18:539. doi: 10.1186/s12885-018-4464-9 (PMC5941371; doi:10.1186/s12885-018-4464-9)
Supplement: Supplementary file 1 — The detailed procedures of GST-pull down assay (DOC 43 kb) [file 12885_2018_4464_MOESM1_ESM.doc]

**The detailed procedures of GST-pull down assay**

**A. Small-scale culture (pilot experiment to check if GST tagged protein is expressed properly)**

1. Growth and induction of cells

a. Inoculate several colonies* containing pGEX-unp-ECD (unplugged extracellular domainexpressing vector) into 2 ml 2x YTA medium (with amp).

b. For comparison, inoculate a control tube with bacteria transformed with the parental pGEX plasmid.

c. Grow the inoculum for 12-15 h at 37 °C with vigorous shaking.

d. Then dilute the culture 1:100 into 2 ml of 2x YTA medium (with amp).

e. Grow liquid cultures to an A600 of 0.6-0.8 (about 3-5 h) with vigorous agitation at 20-37 °C.

f. Induce fusion protein expression by adding 1-10 μl of 100 mM IPTG for each ml of culture volume (final concentration=0.1-1.0 mM).

g. Continue incubation for an additional 1-2 h.

h. Transfer the liquid cultures to Eppendorf tubes.

i. Centrifuge to pellet cells (5 sec in a microcentrifuge) and discard the supernatants. Drain the pellets thoroughly and place tubes on ice.

j. Resuspend each pellet in 50 μl of ice-cold 1x PBS for each ml of culture that centrifuged.

k. Remove 10 μl of these resuspended cells into labeled tubes (for later use in SDS-PAGE analysis).

**2. Cell lysis (freeze/thaw)**

a. Prepare a 10 mg/ml lysozyme solution in water. Add 1 μl of lysozyme solution to each 100 μl of cell suspension.

b. Vortex tubes gently to disperse lysozyme. Allow tubes to incubate at RT for 5 min.

c. In a fume hood, prepare a dry ice bath in an ice bucket by adding dry ice and isoproponal until a slushy consistency is achieved. Prepare warm water bath in a separate ice bucket.

d. Place tubes containing lysozyme-treated cell suspensions in the dry ice bath until cells are frozen solid for about 20 sec.

e. Transfer tubes to a flotation carrier and place in the warm water bath until the suspension becomes fully liquid, about 1 min. Repeat freeze/thaw cycle 10 times.

f. Spin at full speed in a microcentrifuge for 10 min to remove insoluble material. Decant the supernatants into fresh tubes**. Save 10 μl aliquot of the insoluble and soluble material for analysis by SDS-PAGE.

**3. Preparation of glutathione sepharose 4B**

a. Gently shake the bottle of sepharose 4B to resuspend the matrix.

b. Use a pipet to remove sufficient slurry for use and transfer to 15 ml falcon tube. (Dispense 1.33 ml of original sepharose slurry per ml of bed volume required.)

c. Sendiment the matrix by centrifugation at 500 x g for 5 min. Carefully decant the supernatant.

d. Wash the sepharose 4B by adding 10 ml of cold 1x PBS per 1.33 ml of the original slurry of glutathione sepharose dispensed. Invert to mix.

Note: Sepharose 4B must be thoroughly washed with PBS to remove the 20% ethanol storage solution. Residual ethanol may interfere with subsequent procedures.

e. Sediment the matrix by centrifugation at 500 x g for 5 min. Decant the supernatant.

f. For each 1.33 ml of the original slurry, add 1 ml of 1x PBS. This produces a 50% slurry. Mix well prior to the subsequent pipetting steps (Sepharose 4B equilibrated with PBS may be stored at 4 °C for up to a month).

**4. Purification of fusion proteins**

a. Add 20 μl of 50% slurry of glutathione sepharose 4B to each lysate supernatant and mix gently for 5 min at RT.

b. Add 100 μl of 1x PBS, vortex briefly, and centrifuge fro 5 sec to sediment the sepharose beads.

c. Discard the supernatants. Repeat this 1x PBS wash twice for a total of 3 washes.

d. Elute the fusion protein by the addition of 10 μl of glutathione elution buffer. Suspend the sepharose beads and incubate at RT for 5 min.

e. Centrifuge for 5 min to sendiment the sepharose beads, and transfer the supernatants to fresh tubes.

**5. Analysis of fusion proteins**

a. Use 10 μl aliquots of each sample to run the SDS-gel.

b. Stain with Coomassie blue to visualize the parental pGEX and the fusion protein. GST band is 29 kDa.

c. Or, perform western blots using anti-GST antibody.

*The bacteria can be grown on LB+Amp plates, but better be grown on 2x YTA+Amp plates.

**If the lysate is too viscous for handling. Add DNase I to a final concentration of 10 μg /ml in step 9.

**B. Large-scale culture (for GST binding assay)**

**1. Growth and induction of cells**

a. Inoculate a single colony containing a recombinant pGEX-unp-ECD or pGEX into 2 ml 2x YTA medium (with amp).

b. Grow the inoculum for 12-15 h at 37 °C with vigorous shaking.

c. Then dilute the culture 1:100 into desired volume of 2x YTA medium (with amp). (I usually grow 400 ml culture, so I combine two small cultures).

d. Grow liquid cultures to an A600 of 0.6-0.8 (about 2.5-3 h) with vigorous agitation at 37 °C.

e. Induce fusion protein expression by adding the desired volume of 100 mM IPTG to the culture (final concentration=1.0 mM).

f. Continue incubation for an additional 1 h at low temperature (30 °C).

g. Transfer the liquid cultures to labeled centrifuge tubes.

h. Centrifuge to pellet cells (7,700 x g for 10 min) and discard the supernatants. Drain the pellets thoroughly and place tubes on ice.

i. Resuspend each pellet in 50 μl of ice-cold 1x PBS for each ml of culture that centrifuged (20 ml iced 1x PBS for 400 ml cultures).

j. Remove 10 μl of these resuspended cells into labeled tubes (for later use in SDS-PAGE analysis).

**2. Cell lysis (sonication)**

a. Lyse the cells using a sonicator equipped with an appropriate probe.

Note: I usually do lysozyme treatment before sonication (1 μl of lysozyme, 10 mg/ ml in H2O, to 100 μl of cell suspension), freeze and thaw using dry ice and warm water bath for 3 times.

b. Sonicate 10-20 sec each time and totally 10-15 times.

c. Lysis is complete when the cloudy cell suspension becomes translucent. The frequency and intensity of sonication should be adjusted such that complete lysis occurs in 10 sec without frothing (which can denature proteins). The extent of lysis maybe monitored by microscopic examination of culture sonicates.

d. Large-scale sonicates may require the addition of 20% TritonTM X-100 to a final.

e. Centrifuge at 8,000 rpm for 10 min to remove insoluble material. Transfer the supernatants to fresh tubes. Save a 10 μl aliquot of the insoluable material for SDS-PAGE.

**3. Preparation of glutathione sepharose 4B**

a. Gently shake the bottle of sepharose 4B to resuspend the matrix.

b. Use a pipet to remove sufficient slurry for use and transfer to an 15 ml falcon tube (dispense 1.33 ml of original sepharose slurry per ml of bed volume required).

c. Sendiment the matrix by centrifugation at 500 x g for 5 min. Carefully decant the supernatant.

d. Wash the sepharose 4B by adding 10 ml of cold 1x PBS per 1.33 ml of the original slurry of glutathione sepharose dispensed. Invert to mix. (Sepharose 4B must be thoroughly washed with PBS to remove the 20% ethanol storage solution. Residual ethanol may interfere with subsequent procedures).

e. Sediment the matrix by centrifugation at 500 x g for 5 min. Decant the supernatant.

f. For each 1.33 ml of the original slurry, add 1 ml of 1x PBS. This produces a 50% slurry. Mix well prior to the subsequent pipetting steps (sepharose 4b equilibrated with PBS may be stored at 4 °C for up to a month.

**4. Purification of fusion proteins**

a. Add 2 ml of a 50% slurry of glutathione sepharose 4B equilibrated with 1x PBS to each 100 ml of sonicates (400 μl of 50% slurray to 20 ml sonicates).

b. Incubate with gentle agitation at RT for 1 h.

c. Centrifuge at 500 x g (1,400 rpm) for 5 min. Remove the supernatant and save a small volume for analysis by SDS-PAGE to measure the efficiency of binding to the matrix.

d. Wash the matrix with 10 bed volumes of 1x PBS (2 ml 1x PBS for 400 μl 50% slurray). Centrifuge at 500 x g for 5 min. Remove the supernatant. Repeat twice more for a total of three washes.

e. In the last wash or after 3 washes, the lysis buffer* in which the pull-down experiment will be performed can be used instead.

f. After last washing extensively, add the lysis buffer to the GST-fusion protein/sepharose 4B beads, resulting a 50% slurry. Keep the beads on ice. (can add 100 μg ml-1 BSA to reduce the nonspecific binding) (200 μl 1x PBS+PIs).

g. Take 20 μl out for SDS-PAGE gel and the coomassie staining to measure the amount of bound protein. Dilute GST protein in different folds to get similar amounts as for GST fusion protein in order to find the right dilution.

**5. Preparation of Wnt conditional medium**

a. Grow 293T cells and transfect the cells with Wnt expressing construct and incubate for 3-5 days. Wnt protein will be secreted into the medium.

b. Harvest medium and centrifuge at 1,000 x g (2,000 rpm) for 10 min to remove cells.

c. Filter through a nitrocellulose membrane (0.2 microcentimeter).

d. Concentrate the medium through millipore ultra centrifugal fliter (10 kDa).

For a swinging rotor, spin at maximum 4,000 x g for 15-45 min.

For 35 degree fixed angle rotor, spin at maximum 5,000 x g for 15-45 min.

Leave the volume gradations facing up.

For pre-rinse the centrifugal filter, add deionized water and spin for a couple of minutes, don’t leave the membrane dry.

e. Take the concentrate out by using the thin-end tip. and add lysis buffer to 1 ml.

**6. Preclear the conditioned medium**

a. Preclear the medium by adding about 15 μl of undiluted GST beads to 1 ml medium and incubate at 4 °C for 2 h.

(For both control medium and wnt11rFLAG medium.)

**7. Pull-down experiments**

a. Add 20 μl of GST-SV1-ECD beads to 1 ml precleared medium.

Incubate at 4 °C for 2 h to O/N. (totally 2-5 μg of fusion protein**).

Dilute GST-beads with naked beads appropriately (to control the same amount of input for GST and GST fusion protein). And add the same amount of beads to the medium.

b. Centrifuge at 500 x g for 5 min and remove the supernatants.

c. Wash the beads using lysis buffer* for 3-4 times. e.g. 50 μl of beads need 1 ml lysis buffer per wash.

200 μl lysis buffer for 20 μl beads.

d. Add 2x SDS loading buffer to the beads and then boil for 10 min. e.g. 50 μl of beads need 30-40 μl loading buffer.

**8. Analysis of fusion proteins**

a. Use 10 μl aliquots of each sample to run the SDS-gel. Stain with Coomassive blue to visualize the parental pGEX and the fusion protein. GST protein is 29 kDa.

determine the amount of protein bound to the beads per volume.

b. Take 20 μl of beads out (from step 20) and then eluted with loading buffer, boil and then run SDS-PAGE gel. As a maker, run BSA with different amounts alongside. Stain the gel with G-blue and then determine the amount of protein attached to the beads per 20 μl volume.
