# Supplementary figures and images for: Histone methyltransferase SETDB1 promotes cells proliferation and migration by interacting withTiam1 in hepatocellular carcinoma
Source: BMC Cancer. 2018 May 8;18:539. doi: 10.1186/s12885-018-4464-9 (PMC5941371; doi:10.1186/s12885-018-4464-9)

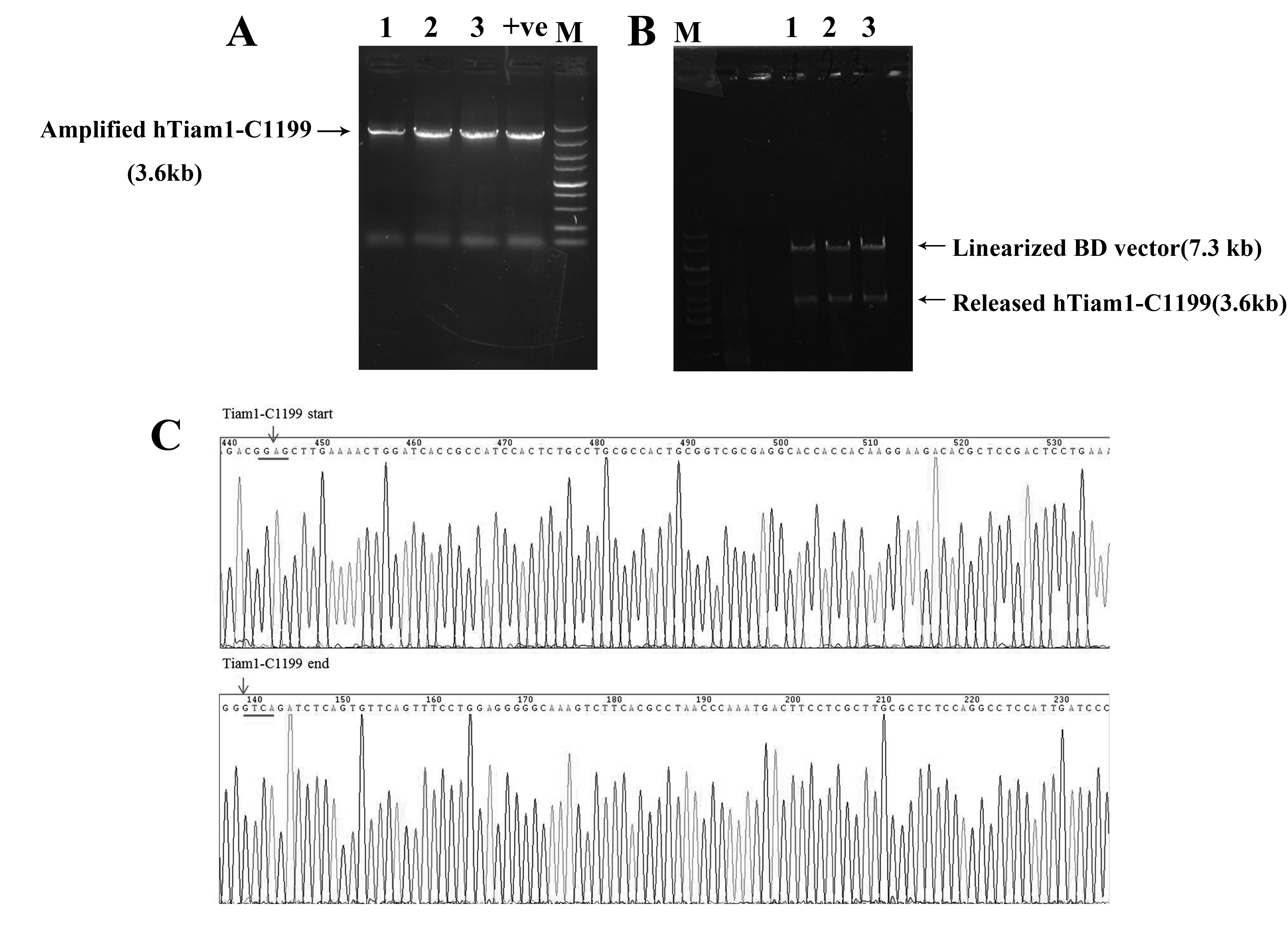

Supplement: Supplementary file 2 — Figure S1. A. Confirmation of the BD-hTiam1-C1199 clones by PCR M:5000bpDNA; Ladder one to three: three BD-hTiam1-C1199 clones amplified by hTiam1-C1199 forward and reversed primers; +ve: commercial human Tiam1 clone amplified by hTiam1-C1199 forward and reversed primers. B Confirmation of the BD-hTiam1-C1199 clones by enzyme digestion. M:1 kb DNA; Ladder one to three: three BD-hTiam1-C1199 clones digested with NdeI and XmaI.). C Partial sequencing map of Tiam1-C1199 clone. (TIF 13173 kb) [file 12885_2018_4464_MOESM2_ESM.tif]

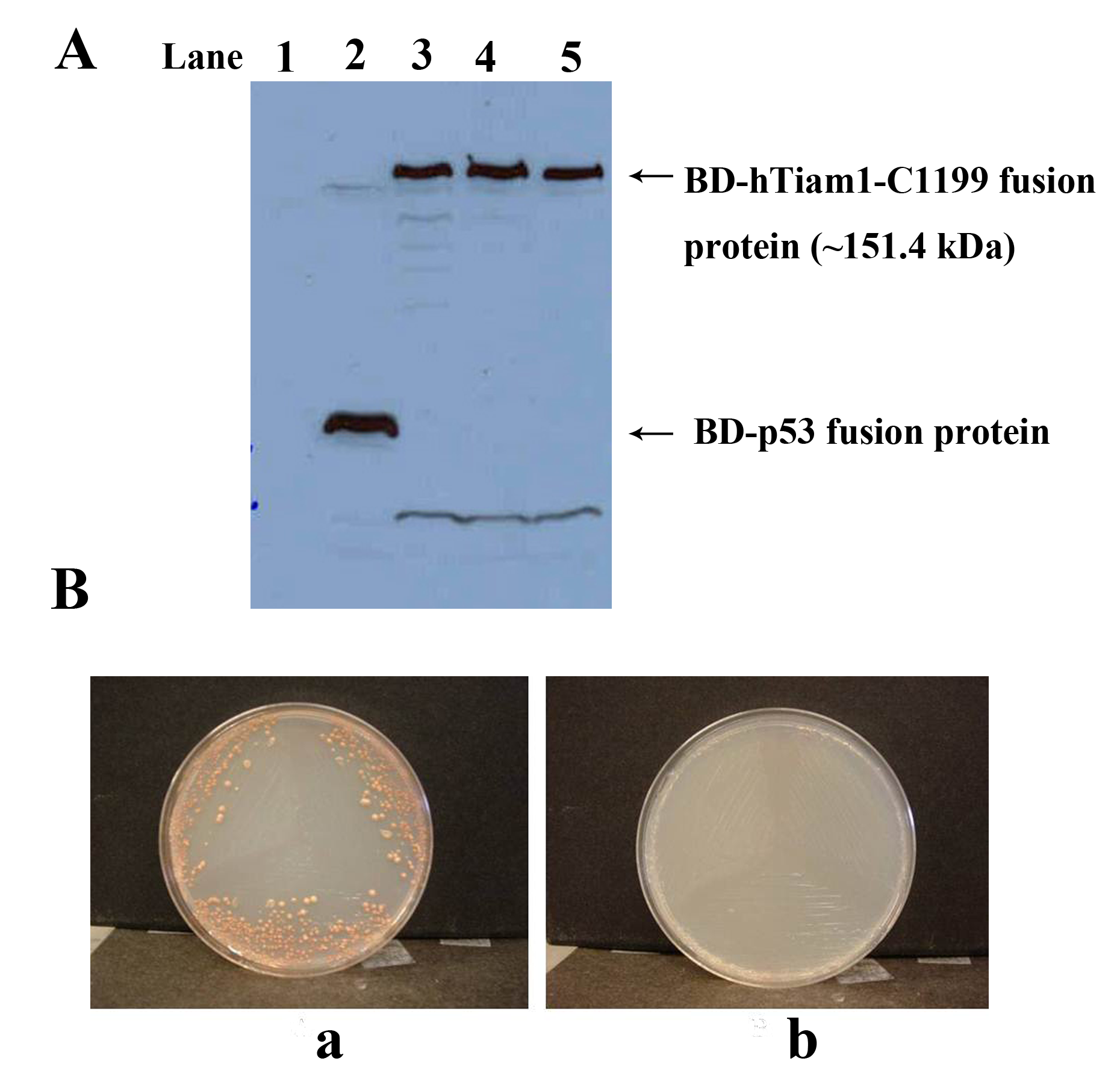

Supplement: Supplementary file 3 — Figure S2. (A) Expression of the BD-hTiam1-C1199 clones detected by anti-c-Myc antibody. (ladder1:BD alone, ladder1 2: BD-p53 fusion protein, ladder3-5: BD-hTiam1-C1199 clone #1-3). (B)Testing for autoactivation by plating AH109 cells co-transformed with the bait plasmid and the AD vector on SCM-2 and -3 plates. SCM-2, SCM plate lacking leucine and tryptophan; cell growth shows the successful co-transformation; SCM-3, SCM plate lacking leucine, tryptophan and histidine; no cell growth shows no autoactivation of transcription by the BD-hTiam1-C1199 fusion protein. (a. SCM/−Trp-Leu (− 2) Plate;b. SCM/−Leu-Trp-His (− 3) Plate). (TIF 9682 kb) [file 12885_2018_4464_MOESM3_ESM.tif]

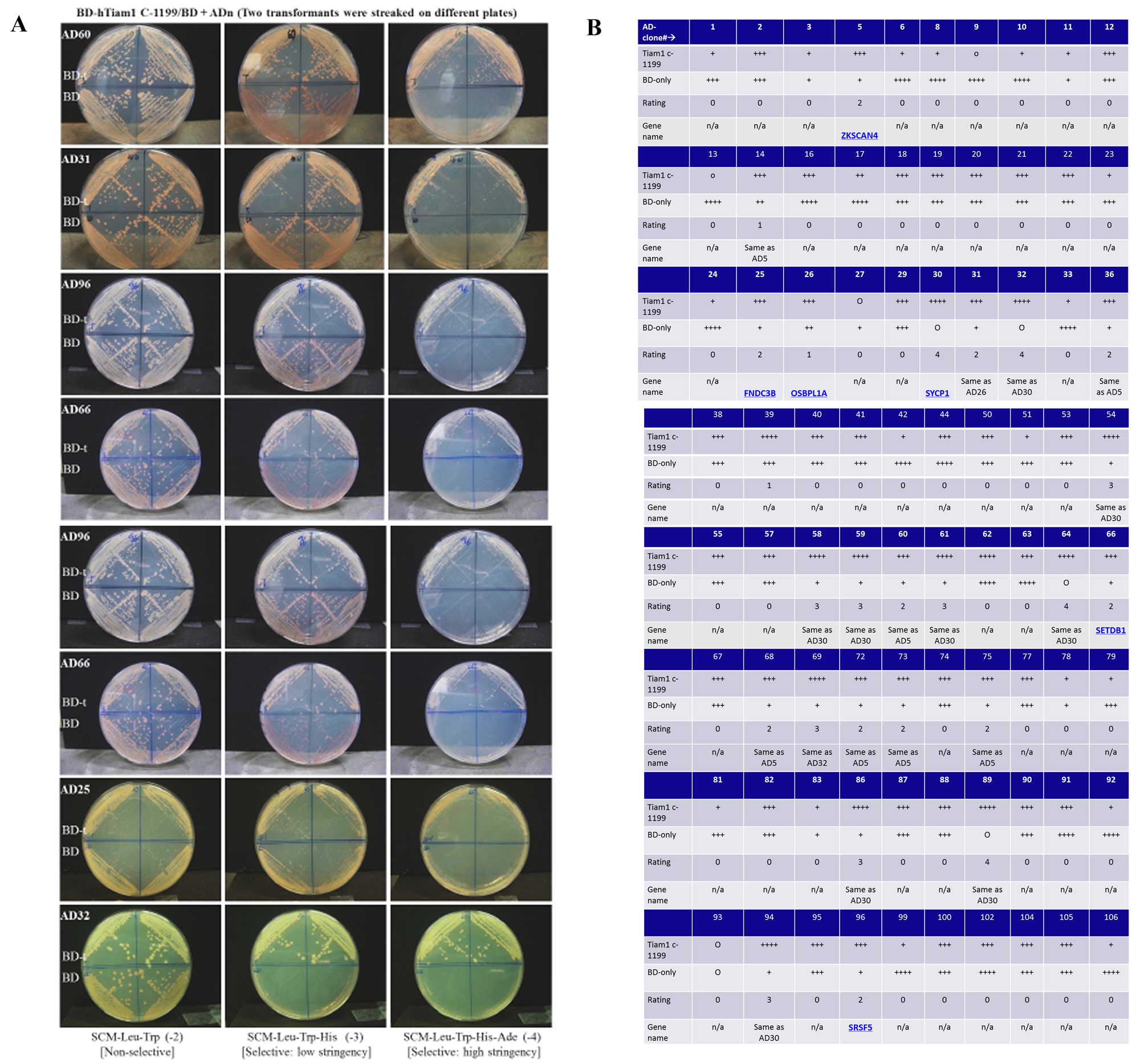

Supplement: Supplementary file 4 — Figure S3. (A) Positive colonies were verified by re-hybrid assay. (B) Summary of Tiam 1 yeast two hybrid results. Rating ≥ 2: Positive candidates; =1: possible candidates (some confirmed); =0:negative candidates (interacting with BD). ‘0’: no colony on SCM-Trp-Leu-His (− 3) plate; ‘+’: small sized and/or red colonies on − 3 only; ‘++’: normal sized white colonies on − 3; ‘+++’: normal sized white colonies on − 3 and small sized and/or red colonies on SCM-Trp-Leu-His-Ade (− 4) plate;++++’ normal sized white colonies on − 4. (TIF 10966 kb) [file 12885_2018_4464_MOESM4_ESM.tif]

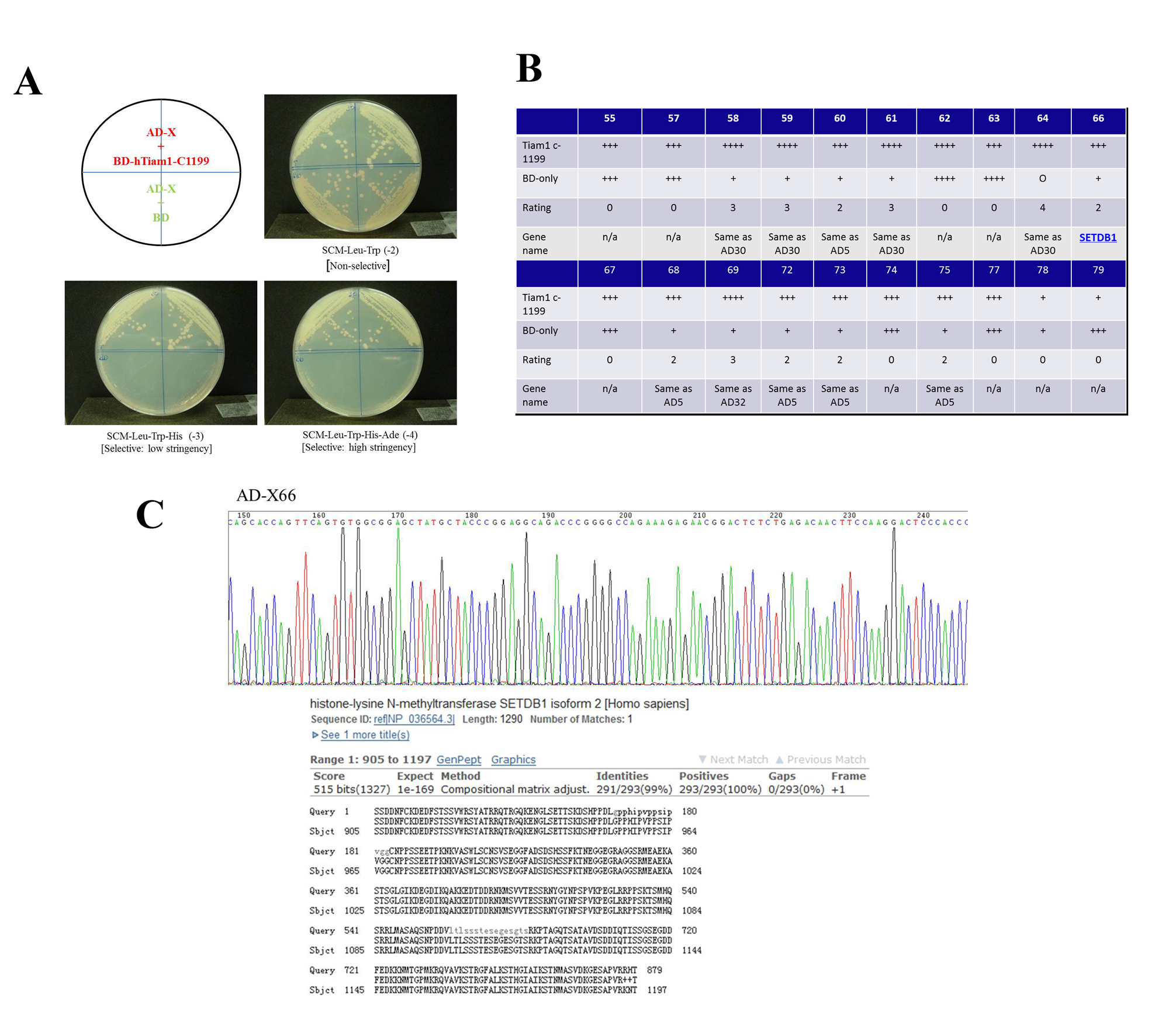

Supplement: Supplementary file 5 — Figure S4. Yeast two-hybrid Screening for proteins interactions with Tiam1 and confirmed by Sequencing and blast NCBI database. A Screening positive clones obtained by using a different degree defective media. B SETDB1was one of the possible protein interaction with Tiam1 by blast NCBI database. C The sequencing of one positive clone screened out. (TIF 10416 kb) [file 12885_2018_4464_MOESM5_ESM.tif]

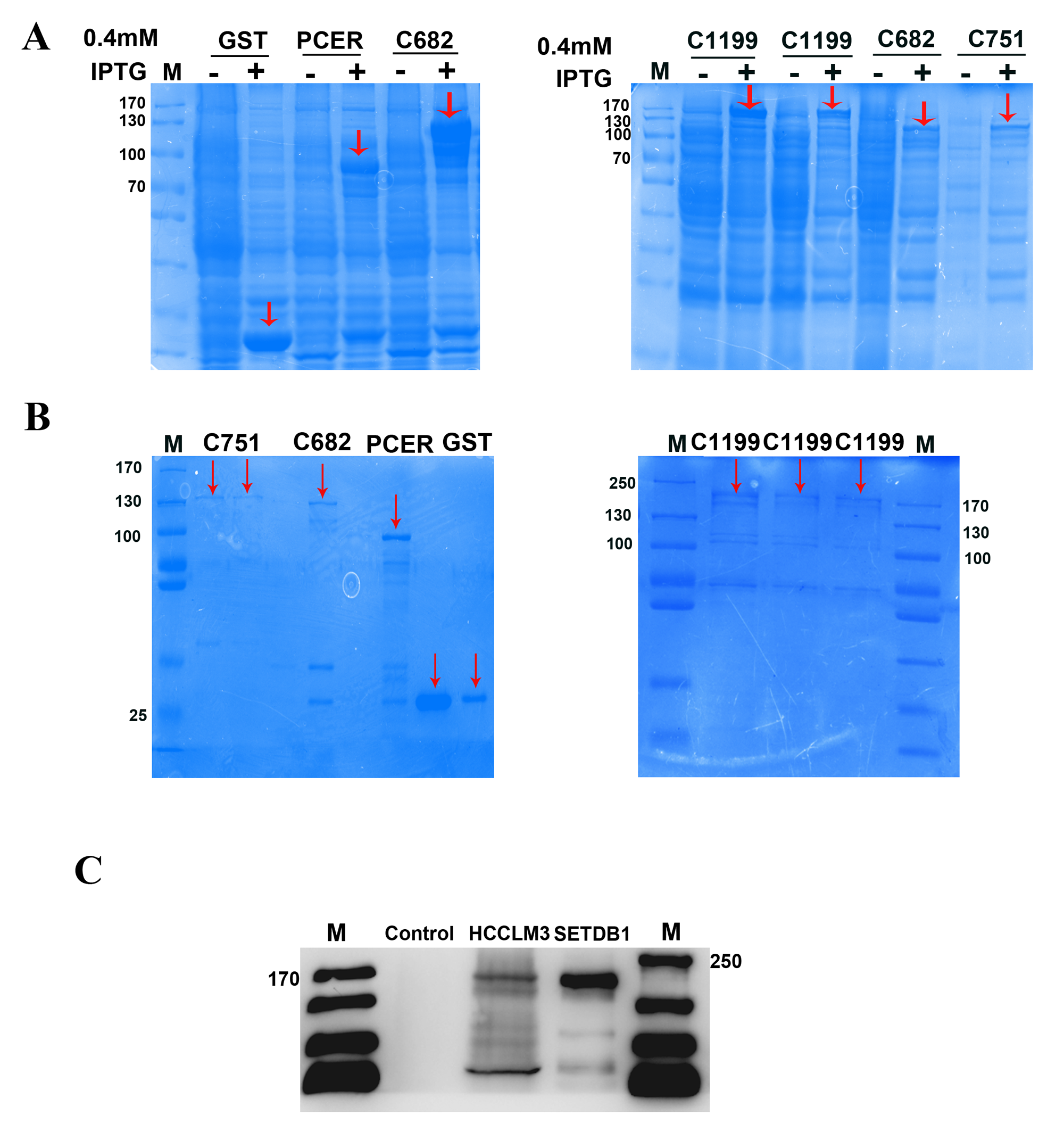

Supplement: Supplementary file 6 — Figure S5. (A) IPTG successfully induction of four different domains as named of Tiam1 and confirmed by Coomassie brilliant blue staining. (B) Four different domains of Tiam1 were purified by agarose beads with GST tag. (C) Verify the expression of SETDB1by TNT transcription and translation kit in vitro. (TIF 12668 kb) [file 12885_2018_4464_MOESM6_ESM.tif]
